# Temporal variation of cesium isotope concentrations and atom ratios in zooplankton in the Pacific off the east coast of Japan

**DOI:** 10.1038/srep39874

**Published:** 2017-01-04

**Authors:** Takahito Ikenoue, Hyoe Takata, Masashi Kusakabe, Natsumi Kudo, Kazuyuki Hasegawa, Takashi Ishimaru

**Affiliations:** 1 Central Laboratory, Marine Ecology Research Institute, 300 Iwawada, Onjuku-machi, Isumi-gun, Chiba 299-5105, Japan; 2 Marine Ecology Research Institute, Tohwa-Edogawbashi Bldg., 347 Yamabuki-cho, Shinjuku-ku, Tokyo 162-0801, Japan; 3 Department of Marine Science, Tokyo University of Marine Science and Technology, 5-7, Konan 4, Minato-ku, Tokyo 108-8477, Japan

## Abstract

After the Fukushima Daiichi Nuclear Power Plant accident in March 2011, concentrations of cesium isotopes (^133^Cs, ^134^Cs, and ^137^Cs) were measured in zooplankton collected in the Pacific off the east coast of Japan from May 2012 to February 2015. The time series of the data exhibited sporadic ^137^Cs concentration peaks in zooplankton. In addition, the atom ratio of ^137^Cs/^133^Cs in zooplankton was consistently high compared to that in ambient seawater throughout the sampling period. These phenomena cannot be explained fully by the bioaccumulation of ^137^Cs in zooplankton via ambient seawater intake, the inclusion of resuspended sediment in the plankton sample, or the taxonomic composition of the plankton. Autoradiography revealed highly radioactive particles within zooplankton samples, which could be the main factor underlying the sporadic appearance of high ^137^Cs concentrations in zooplankton as well as the higher ratio of ^137^Cs/^133^Cs in zooplankton than in seawater.

The Great East Japan Earthquake occurred on 11 March 2011, and the ensuing tsunami resulted in the release of large amounts of radionuclides from the Fukushima Daiichi Nuclear Power Plant (FDNPP) into the atmosphere and ocean^1^,^2^. The main radionuclides discharged were ^131^I, ^134^Cs, and ^137^Cs, as reported by the Tokyo Electric Power Company (TEPCO)^3^. The half-lives of radiocesium, ^134^Cs and ^137^Cs, are 2.07 and 30.17 years, respectively. Due to their longer half-lives than ^131^I (8.04 days), continuous monitoring of the levels of radiocesium contamination is necessary to evaluate the impacts of these radionuclides on marine organisms, which is important for addressing risks to human health through consumption of fisheries resources.

Zooplankton play an important role in the marine biogeochemical cycle as secondary producers in the food web, and are major food resource for fishes and organisms of higher trophic levels. Therefore, it is imperative to study the level and temporal variation of radiocesium in zooplankton in association with seawater, sediment, and suspended marine particles. The result will be of great help to predict the fate of radiocesium in marine ecosystem.

After the FDNPP accident, elevated ^137^Cs concentrations in zooplankton were observed in the western North Pacific^4^,^5^,^6^,^7^. From May 2012 to January 2013, Takata *et al*.^7^ measured radiocesium concentrations in zooplankton collected at sampling locations identical to those used in this study; their findings suggested that the concentration of ^137^Cs in ambient seawater influenced the variations of ^137^Cs in zooplankton. However, Kaeriyama *et al*.^6^ carried out observations 10 months earlier than Takata *et al*.^7^ and noted that the temporal change of ^137^Cs in zooplankton was not synchronous with that of seawater. Based on these findings, they proposed a dynamic non-equilibrium model of ^137^Cs transfer between organisms and the surrounding seawater. The model described the progress of ^137^Cs contamination in zooplankton from the beginning of the FDNPP accident (dynamic non-equilibrium state) to the restoration phase (dynamic equilibrium state).

In this study, we investigated the temporal variation of FDNPP-derived cesium isotopes in zooplankton collected from May 2012 to February 2015. We elucidated the factors controlling the changes in ^137^Cs concentration in zooplankton by utilizing other relevant data such as stable cesium (^133^Cs) and aluminum concentrations, and the taxonomic composition of the zooplankton.

## Results and Discussion

### Abundance and taxonomic compositions of zooplankton

**Figure 1:**
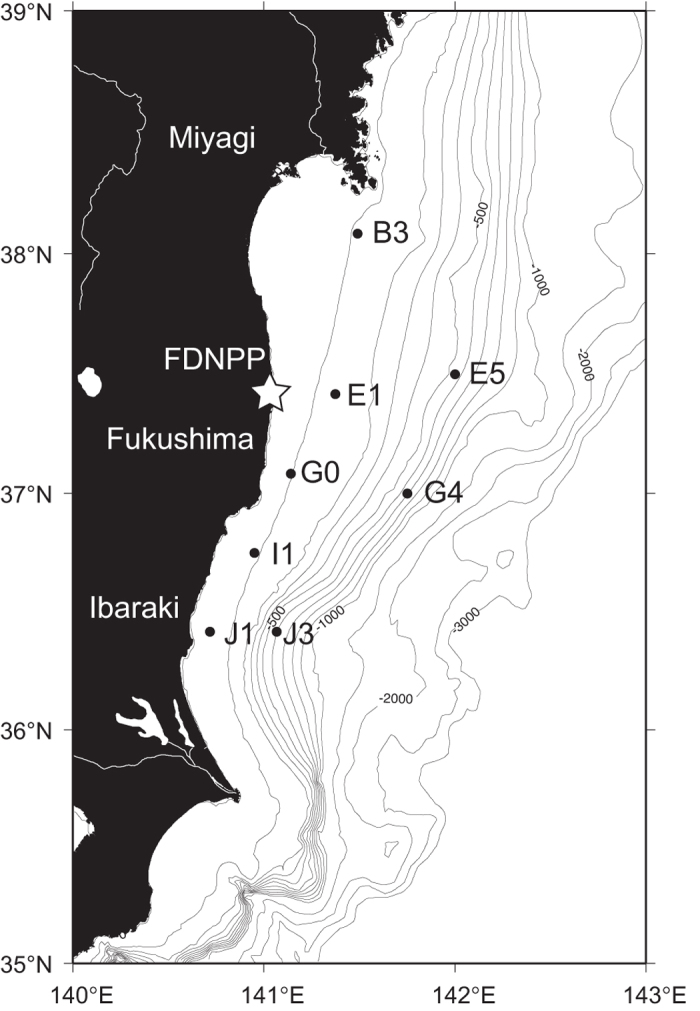
Map of the sampling locations of zooplankton, seawater, and surface sediment. Solid circles indicate sampling stations, and the star marks the Fukushima Daiichi Nuclear Power Plant (FDNPP). The Map is created online at http://sfb574.geomar.de/gmt-maps.html 8.

**Figure 2:**
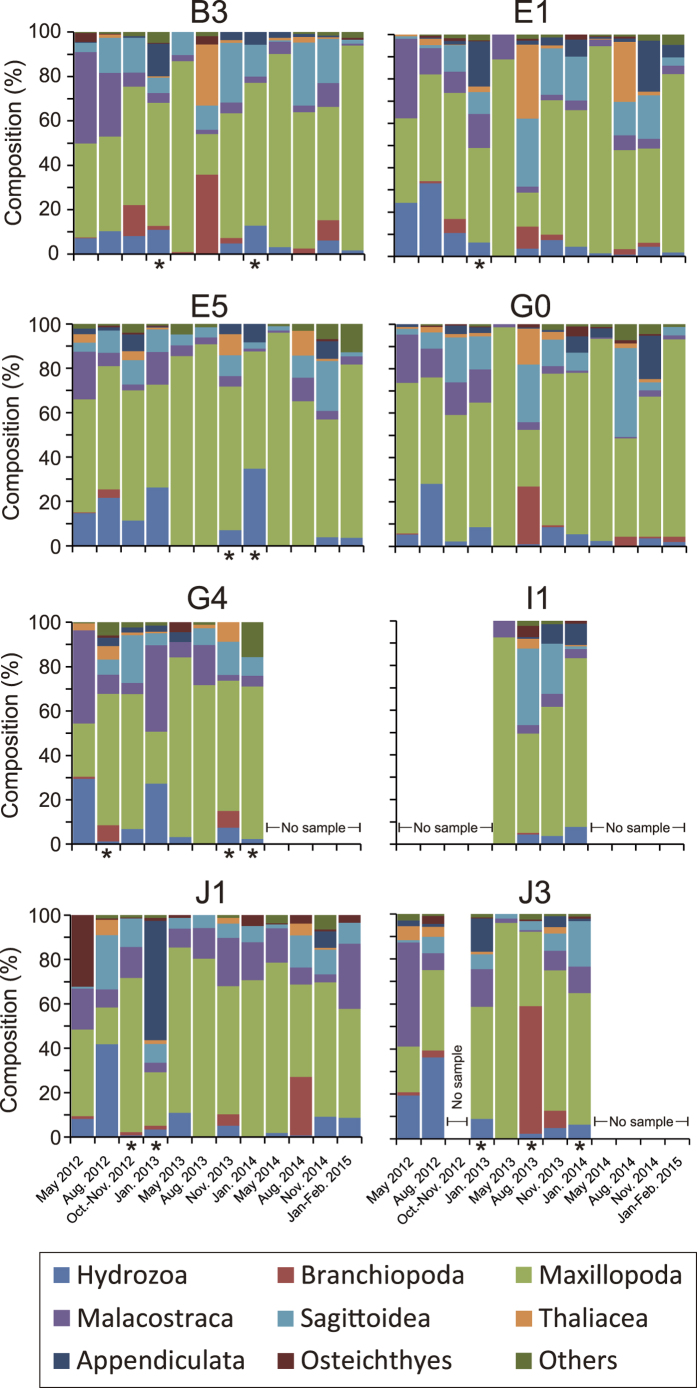
Temporal variation of class-level taxonomic compositions of zooplankton. The samples containing a portion of phytoplankton are marked with an asterisk.

Zooplankton samples were collected at eight sampling sites in the Pacific off the east coast of Japan, where the radioactivity levels of seawater and surface sediments have been monitored under contract with the Japanese Ministry of Education, Culture, Sports, Science and Technology (2011–2013) and the Secretariat of the Nuclear Regulation Authority (2013-present) (Fig. 1). Seventy-nine samples were collected from May 2012 to February 2015. Table S1 summarizes the data on the plankton samples. Zooplankton biomass varied by two orders of magnitude, ranging from 1.1 to 562.8 mg-wet/m^3^, with water content ranging from 82% to 95%. The temporal variation of zooplankton abundance at the class level is shown in Table S1 and Fig. 2. Although the taxonomic composition varied seasonally and geographically, members of Maxillopoda were generally dominant throughout the study. Malacostraca increased from May 2012 to January 2013 at several stations. Appendiculata usually showed low relative abundance but increased in November or January, except at stn. G4. Branchiopoda increased in August 2013 at stns. B3, G0, and J3 and in August 2014 at stn, J1. Thaliacea increased in August 2013 at stns. B3, E1, and G0 and in August 2014 at stn. E1. The taxonomic compositions at stn. J1 in May 2012 (Osteichthyes dominant) and January 2013 (Appendiculata dominant) were especially different from those of the other stations. Osteichthyes was mainly composed of eggs of fishes in this study. It should be noted that thirteen of the samples were replete with microplankton, mainly chain-forming phytoplankton (see asterisks in Fig. 2).

### Temporal variation of radiocesium (^134^Cs and ^137^Cs) and the stable isotope (^133^Cs) in zooplankton

**Figure 3:**
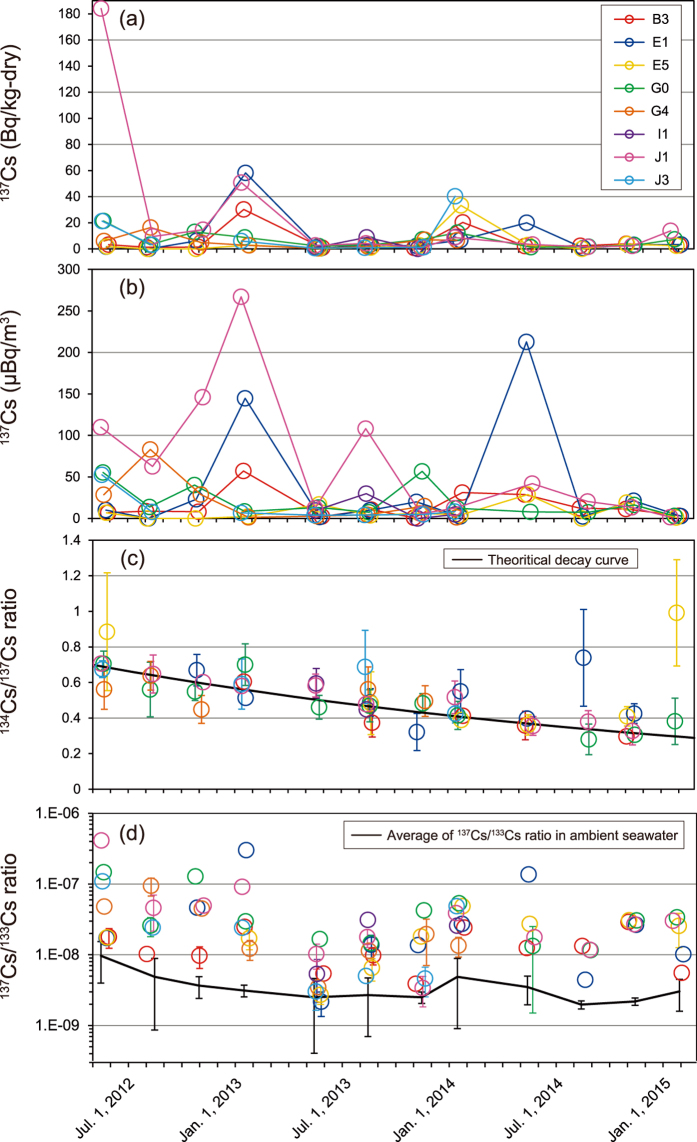
(**a**) Temporal variation of ^137^Cs concentration in zooplankton. The data from May 2012 to January 2013 were from Takata *et al*.^7^. Errors of the data based on 1σ counting statistics are less than or equal to the size of the symbol. (**b**) Temporal variation of ^137^Cs in zooplankton per unit volume of seawater. (**c**) Temporal variation of ^134^Cs/^137^Cs activity ratios in zooplankton. The black line indicates theoretical decay curves for ^134^Cs/^137^Cs activity ratios with an initial ratio of 1 on 11 March 2011. (**d**) Temporal variation of ^137^Cs/^133^Cs ratio in zooplankton. The black line indicates the average ^137^Cs/^133^Cs ratio in ambient seawater for each sampling period. The error bars indicate the standard deviations of the ^137^Cs/^133^Cs ratio in ambient seawater during each sampling period.

Temporal variation of radiocesium (^134^Cs and ^137^Cs) and the stable isotope (^133^Cs) in zooplankton are summarized in Fig. 3. Concentrations of ^134^Cs and ^137^Cs in zooplankton samples and relevant data for May 2013 to February 2015 are summarized in Table S2 (those for May 2012 to January 2013 were reported previously^7^). The concentrations of ^137^Cs in zooplankton varied from 0.26 to 184 Bq/kg-dry during the study period (Fig. 3a); the maximum concentration was observed in May 2012 at stn. J1. Although the concentrations generally seem to have peaks in winter (i.e., January in 2013 and 2014), the temporal pattern at each station showed additional, sporadic concentration peaks as well. That is, relatively high concentrations of ^137^Cs (i.e., >20 Bq/kg-dry) were observed in May 2012 at stns. G0, J1, and J3; in January 2013 at stns. B3, E1, and J1; in January 2014 at stns. B3, E5, and J3; and in May 2014 at stn. E1. In January and February 2015, after nearly 4 years had passed since the accident, the concentration ranged from 2.29 to 13.8 Bq/kg-dry, still one to two orders of magnitude larger than those detected before the FDNPP accident (0.09–0.4 Bq/kg-dry, Kaeriyama *et al*.^9^).

The concentrations of ^137^Cs associated with zooplankton per unit volume of seawater are plotted in Fig. 3b. The highest ^137^Cs concentrations in zooplankton (>100 μBq/m^3^) were observed at stns. E1 and J1. The concentration peaks on a volume basis appeared roughly at the same time as those on a dry-weight basis, although with different magnitudes.

The activity ratios of ^134^Cs/^137^Cs in zooplankton generally decreased with time following a theoretical decay curve with the initial value of the ratio set to 1 on 11 March 2011 (Fig. 3c), indicating that a majority of the radiocesium in zooplankton samples was of FDNPP origin. The two notable data that deviated from the curve (i.e., stn. E1 in August 2014 and stn. E5 in January 2015) were probably attributable to the large errors due to poor counting statistics in the concentrations of ^134^Cs.

**Figure 4:**
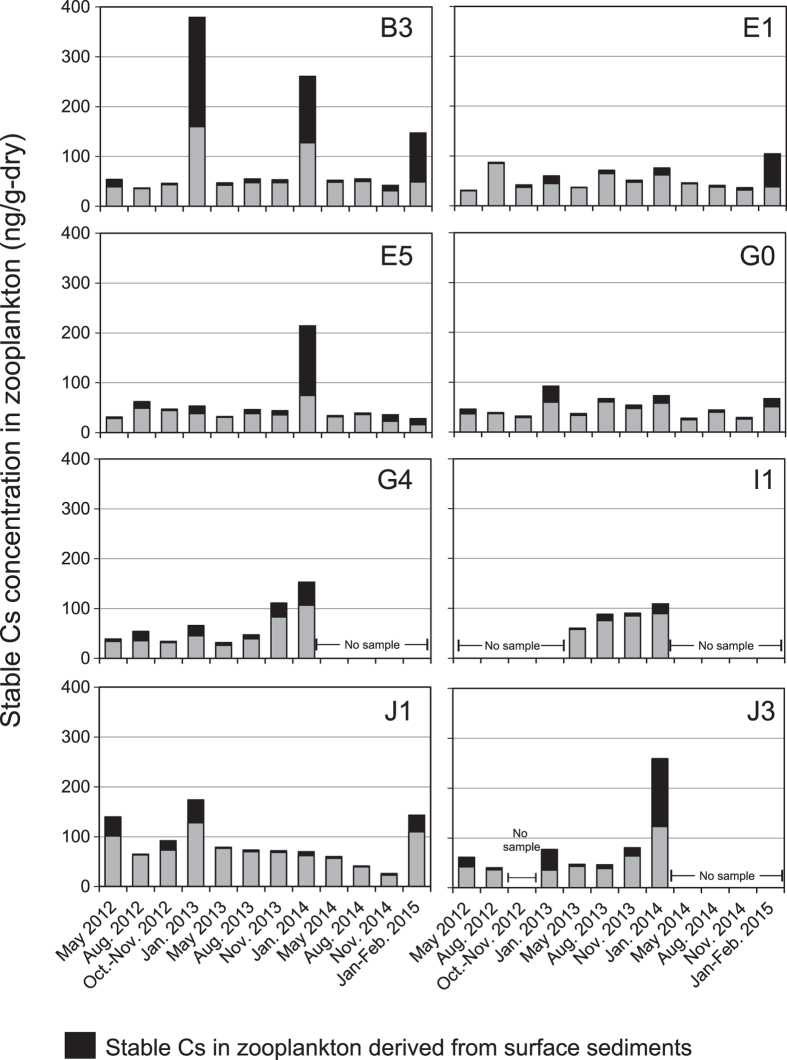
Temporal variation of ^133^Cs concentration in zooplankton. Black bars represent concentrations of ^133^Cs in zooplankton derived from resuspended sediments.

Concentrations of the stable isotope of cesium, ^133^Cs, in zooplankton are given in Table S3 and Fig. 4. The concentration of ^133^Cs ranged from 26 to 379 ng/g-dry, with an average and standard deviation of 72 ± 59 ng/g-dry, from May 2012 to February 2015. While most of them fell within the range of 26–100 ng/g-dry, ^133^Cs concentrations >200 ng/g-dry were observed at stns. B3, E5, and J3 in January. The concentrations of ^133^Cs in zooplankton showed, thus, notable peaks only in winter (Fig. 4). Zooplankton ^133^Cs concentrations were measured before and after the accident in the waters around Japan^6^,^10^,^11^ (Table S4). Although the concentrations varied by an order of magnitude, their range and average were similar to those obtained in this study (Table S4).

### ^137^Cs in zooplankton and its relation to ambient seawater

From May 2012 to January 2013, the average ^137^Cs concentrations in ambient seawater decreased from 9.8 to 3.1 mBq/L and those in zooplankton per unit volume of seawater decreased synchronously, with a correlation coefficient (*r*) of 0.77 between them^7^. However, the relationship did not hold for the entire sampling period in this study (*r* = 0.26). Kaeriyama *et al*.^6^ found a similar trend in the relationship between ^137^Cs in zooplankton and seawater. Here, ratios of ^137^Cs in zooplankton to that of seawater (aCR) are calculated for the comparison of their temporal trends following the notation of Kaeriyama *et al*.^6^. Note that the concept of a concentration ratio (CR) recommended by the IAEA^12^ cannot be applied to the area of this study because neither concentrations have yet stabilized since the FDNPP accident. The aCRs of ^137^Cs ranged from 13 to 2957 L/kg-wet, with an average and standard deviation of 207 ± 395 L/kg-wet from May 2012 to February 2015 (Table S2). They are largely fluctuated and most of them were higher than the CR value of 40 reported by the IAEA (2004). Previous field data and model results after the FDNPP accident^6^,^13^,^14^ also reported large fluctuation of aCRs approximately ranging from 0.2 to 1300 spatiotemporally in various conditions. However, the aCRs of ^137^Cs in January and February 2015, four years after the accident, still showed wide range (from 76 to 350 L/kg-wet, with an average and standard deviation of 165 ± 108) even though the ^137^Cs concentrations in ambient seawater have been reduced and relatively constant to be an average and standard deviation of 3.1 ± 1.0 mBq/L. Therefore, the large fluctuation of aCRs might be influenced by a factor other than bioaccumulation of ^137^Cs in zooplankton via ambient seawater.

Concentrations of ^133^Cs in ambient seawater are shown in Table S3. They were relatively constant, ranging from 307 to 329 ng/L, with an average and standard deviation of 316 ± 7 ng/L. These values are comparable to the concentration of ^133^Cs in Pacific seawater (306 ng/L^15^).

In principle, the ^137^Cs/^133^Cs atom ratio in both seawater and zooplankton should reach to the same value under the pre-accident steady-state situation because of the similarity in biological half-lives of ^137^Cs and ^133^Cs. Although the ratios in zooplankton fluctuate by an order of magnitude spatiotemporally, like the ratios in seawater they seem to show a declining trend (Fig. 3d). However, the latest data (5.5 × 10^−9^ to 3.4 × 10^−8^) for the zooplankton in January and February 2015 were still one order of magnitude larger than those in seawater. In addition, they are higher than those in zooplankton before the FDNPP accident (2.7 × 10^−9^ ± 2.0 × 10^−9^; see Tateda^11^).

The ^137^Cs/^133^Cs ratio in zooplankton should have been controlled by the biological half-life of cesium in plankton, the life span of plankton, and temporal change of the ratio in seawater. If the life span of zooplankton and/or the biological half-life in zooplankton were comparable to or longer than the period of this study (~3 years), and the ratio in seawater decreased significantly over those 3 years, then the ^137^Cs/^133^Cs ratios in the zooplankton would have been higher than those in ambient seawater. However, this was not the case, as the life span of zooplankton is fairly short. Indeed, our samples included *Oikopleura dioica* (Appendiculata), which lives for only 5 days^16^; the longest living species in the samples is *Eucalanus bungii* (Maxillopoda) with a 2-year life span^17^. The ^137^Cs/^133^Cs ratio in ambient seawater ranged from 10^−9^ to 10^−8^ during the 3-year study period. Thus, the discrepancy of the ^137^Cs/^133^Cs ratio between seawater and zooplankton cannot be explained by ^137^Cs accumulating in zooplankton in the preceding period when the seawater was more polluted.

### ^137^Cs in zooplankton and its relation to taxonomic composition

Before the FDNPP accident, Kaeriyama *et al*.^9^ showed clear relationship between the ^137^Cs in zooplankton and taxonomic composition. However, several previous studies found no clear relationship after the accident^5^,^7^. In this study, zooplankton biomass (mg-dry/m^3^) was not correlated with ^137^Cs in zooplankton per unit volume of seawater (μBq/m^3^) (*r* = 0.14), so we examined ^137^Cs in zooplankton and its relation to taxonomic composition. Maxillopoda were generally dominant, but their relative abundance was not correlated with ^137^Cs in zooplankton per unit volume of seawater. Among the eight classes of zooplankton, only the relative abundance of Appendiculata showed a correlation (*r* = 0.51, *p* = 0.000003, *N* = 74) with ^137^Cs in zooplankton per unit volume of seawater. Kaeriyama *et al*.^9^ reported that abundance of gelatinous zooplankton (Hydrozoa, Sagittoidea, Thaliacea, and Appendiculata) probably led to a higher concentration of ^137^Cs. Our results were partially concurrent with this idea, but the timing of the increase in gelatinous zooplankton (except Appendiculata) did not correspond with that of high ^137^Cs in zooplankton. Appendicularians are filter feeder living inside an extracellular, gelatinous house, which enables them to feed on particles down to about 0.2 μm^18^. This efficient filter feeding may sometimes contribute to a high concentration of ^137^Cs in zooplankton. However, no matter how cesium were taken by or eliminated from plankton, ^137^Cs/^133^Cs ratio in the plankton should have converged to that in the ambient seawater in the long run (i.e., longer than a life span of plankton) if the ratio in seawater has been constant for the period. Indeed, the concentrations of ^137^Cs in ambient seawater and accordingly ^137^Cs/^133^Cs ratio as well have been lowered and relatively constant for last over 3 years which is significantly longer than the life span of the zooplankton (at most 2 years). Therefore, zooplankton taxonomic composition could not fully explain the discrepancy of the ^137^Cs/^133^Cs ratio between seawater and zooplankton unless ingested particles contained higher amount of ^137^Cs than ^133^Cs.

**Figure 5:**
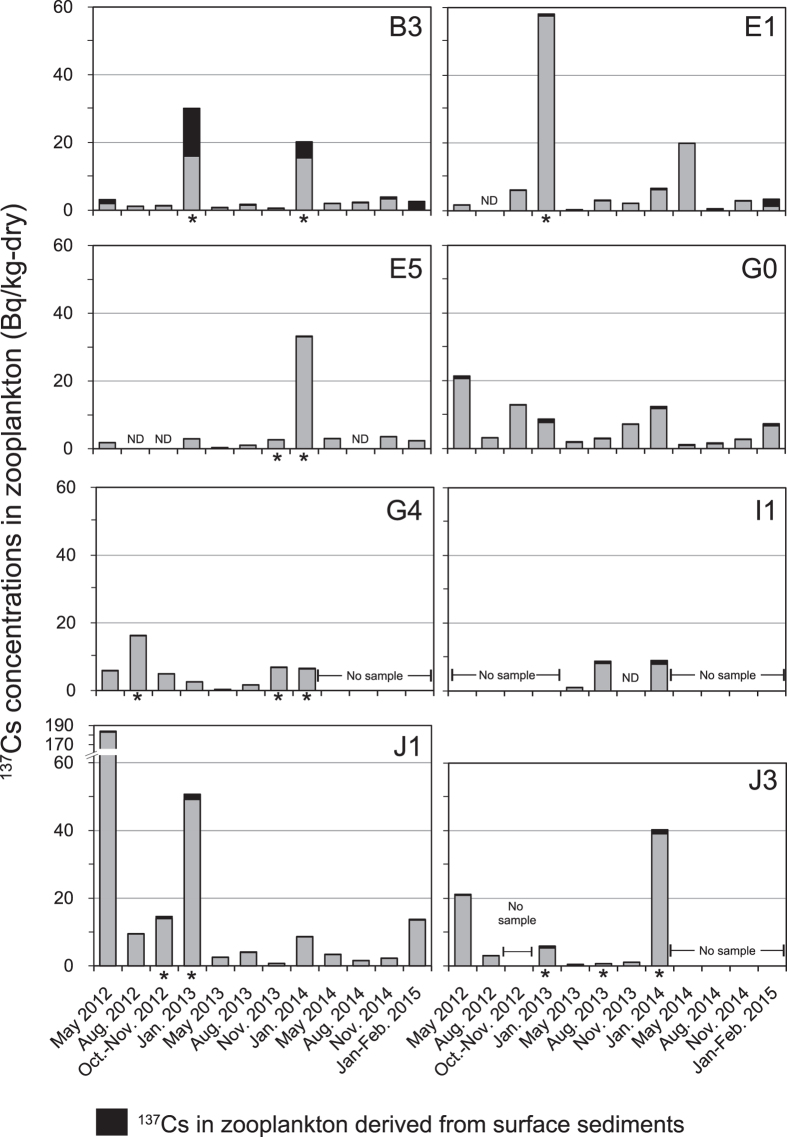
Temporal variation of ^137^Cs concentrations in zooplankton. Black bars represent concentrations of ^137^Cs in zooplankton derived from resuspended sediments. Zooplankton samples containing a portion of phytoplankton are marked with an asterisk.

On the other hand, zooplankton samples that were replete with phytoplankton also tended to have higher ^137^Cs concentrations (Fig. 5). Recently, Baumann *et al*.^13^ found higher ^137^Cs concentrations in marine phytoplankton-rich suspended particles than in zooplankton samples. Thus, the inclusion of phytoplankton could have been a substantial source of ^137^Cs in the zooplankton samples. However, the samples with high concentrations of ^137^Cs reported by Baumann *et al*.^13^ contained not only phytoplankton but also mixed particles. Furthermore, the CR of ^137^Cs in phytoplankton has generally been low in previous studies^12^,^19^. Therefore, it is questionable whether the main source of ^137^Cs was abundant phytoplankton.

### Contribution of resuspended bottom sediment to ^137^Cs in zooplankton

Zooplankton should take ^137^Cs into and/or onto their bodies from the surrounding environment, including seawater, resuspended bottom sediment, and food, so that the variations in these environmental sources may have been related to the spatiotemporal variation of ^137^Cs concentrations in zooplankton. Resuspended sediments are transported horizontally over the continental shelf and slope off Fukushima and adjacent prefectures^20^. Since the concentrations of ^137^Cs in surface sediments have been reported to be higher than those of seawater on a weight basis^21^,^22^, the resuspended bottom sediment could be one of the important source for ^137^Cs in the zooplankton sample, especially in shallow water on the continental shelf and slope, as in this study (Fig. 1). The resuspended sediment is supposed to be incorporated to the zooplankton sample during sample collection and to lesser extent by sediment consumption in plankton body. It is fairly possible to catch resuspended sediment due to the large volume of seawater filtered through the net during sample collection (9414 to 44986 m^3^, see Table S1). For now, we consider the case of sampling artifact on incorporation of resuspended sediment in the zooplankton samples as follows.

The concentrations of ^133^Cs and ^137^Cs in zooplankton sample derived from resuspended sediment (^133^Cs_pl (sed)_ and ^137^Cs_pl (sed)_) can be estimated based on the following two assumptions.

Assumption 1: Aluminum in zooplankton sample was only derived from resuspended sediment because the dissolved aluminum concentration in seawater is negligibly small compared to that in surface sediments^23^.

Assumption 2: Cesium in the zooplankton sample was derived from two sources, one from seawater and the other from surface sediment. Cs/Al ratio in zooplankton sample derived from resuspended surface sediments is equal to the ratio in the surface sediment.

We used the following equations for the estimations of sediment-derived ^133^Cs and ^137^Cs in zooplankton:


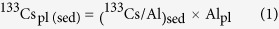



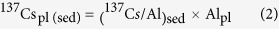


where (^133^Cs/Al)_sed_ and (^137^Cs/Al)_sed_ are concentration ratios of ^133^Cs and ^137^Cs to aluminum in sediments on a weight basis and Al_pl_ is the concentration of aluminum in zooplankton. Because the concentrations of ^133^Cs were not determined for the sediments collected before May 2014, we calculated the average values of the ^133^Cs/Al ratio of sediments collected from May 2014 to February 2015 independently for each station and applied them as representative values for each station.

The concentrations of ^133^Cs and ^137^Cs derived from resuspended sediment in zooplankton are summarized in Figs 4 and 5 and Table S5. Relatively high concentrations of ^133^Cs (>200 ng/g-dry) observed at stns. B3, E5, and J3 in January correspond with the time when the contributions of resuspended sediment to ^133^Cs in zooplankton were >50% (Fig. 4). These facts indicate that resuspended sediments could influence ^133^Cs concentration in zooplankton in winter, when the resuspension of surface sediments is expected to occur by vigorous vertical mixing. The resuspended sediment contribution to ^137^Cs in zooplankton, however, was not a main factor in the increase of ^137^Cs concentration in zooplankton; the contribution was 6.0 ± 11.0% on average except for stn. B3, which had higher contribution of 25.5 ± 24.8% (Fig. 5). It is interesting to know that the abundance of ^137^Cs in the surface sediment at site B3 have been temporally decreasing with the highest rate in the monitoring area, indicative of vigorous sediment resuspension (Kusakabe *et al*., in preparation).

In addition to the sampling artifact on incorporation of resuspended sediment in zooplankton samples, there would be a sediment consumption in plankton body. In this case, equations (1) and (2) cannot be applied to the conditions that all the sediment-derived aluminum and cesium are consumed into the plankton body and they behave differently inside the plankton body with different biological half-lives. Resuspended sediment particles are usually composed of mineral fraction and organic fraction. As for sediment mineral fraction, Cs/Al ratio of resuspended sediment particle in the plankton sample does not change no matter whether sediment particle is in or on the plankton body because aluminum in sediment is the highly immobile, main component of clay mineral, which fixes cesium irreversibly^24^,^25^,^26^. Therefore, cesium and aluminum derived from sediment mineral fraction are not solubilized in the plankton body, that means apparent biological half-life of aluminum is equal to that of cesium derived from resuspended sediment particle. As for sediment organic fraction, it can be source of the dissolved cesium in the body of plankton via decomposition and/or ion exchange. Ono *et al*.^27^ reported a high affinity of radiocesium for organic fraction in marine sediments, implying that cesium may have been taken into the bodies of zooplankton. In this case, sediment organic fraction could change Cs/Al ratio in the bodies of zooplankton. However, the average of inventory ratio of the radiocesium in the sediment organic fraction in offshore-Fukushima region was estimated to be 5.7 ± 3.0%^27^, which was fairly lower than that in the sediment mineral fraction. In addition, the inventory of aluminum in the sediment is not changed by dissolution of the sediment organic fraction since the organic fraction doesn’t contain aluminum. Therefore, even if all the organic fraction were dissolved in the plankton body, the errors in the estimation of sediment-derived cesium introduced by assumptions 1 and 2 would be at most 10%. Thus, the elevated values of ^137^Cs/^133^Cs ratio in zooplankton could not be ascribed significantly to incorporation of resuspended bottom sediment into zooplankton.

### ^137^Cs-enriched particles in zooplankton samples

**Figure 6:**
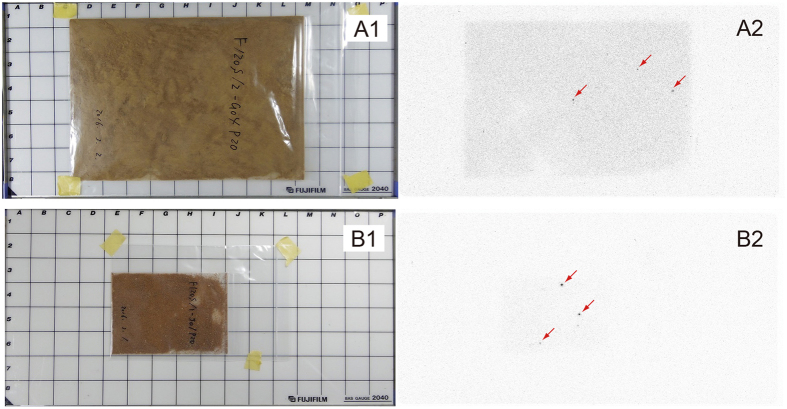
(**A1**) Dried zooplankton sample in a plastic zipper bag (collected at stn. G4 in August 2012). (**A2**) Autoradiograph of A1. (**B1**) Dried zooplankton sample in a plastic zipper bag (collected at stn. J1 in May 2012). (**B2**) Autoradiograph of B1. Highly radioactive particles are indicated by red arrows.

As shown above, there needs to be an additional input of ^137^Cs to zooplankton to account for the persistently higher concentrations of ^137^Cs and ^137^Cs/^133^Cs ratios in zooplankton than those before the FDNPP accident. Studies in the region of the FDNPP have revealed the existence of small, highly radioactive particles in the atmosphere and terrestrial soil^28^,^29^,^30^,^31^. If these particles exist in the seawater as well, they could be a candidate source for additional ^137^Cs in zooplankton. Autoradiography was used to survey such particles in those samples with higher concentrations of ^137^Cs than of the samples, collected at stn. G4 in August 2012 and at stn. J1 in May 2012. Autoradiographic images are shown in Fig. 6. Several spots were recognized on the images, indicating the existence of highly radioactive particles in the samples.

Relatively high concentrations of ^137^Cs were frequently recognized in filter-feeding zooplankton and zooplankton accompanied by abundant phytoplankton. The filter feeders can efficiently catch particles in the micron range. Many aquatic organisms, especially phytoplankton, produce transparent exopolymer particles^32^,^33^, which also can adhere to small particles. The total radioactivity levels of ^137^Cs in zooplankton samples used in this study ranged from 0.02 to 4.83 Bq/sample, with an average and standard deviation of 0.49 ± 0.80 Bq/sample (Table S6). Adachi *et al*.^34^ have estimated that ^137^Cs activity of radioactive particles ranges from 0.66 to 3.27 Bq/particle. If the radioactive particles found in the zooplankton samples have the same activity reported by Adachi *et al*.^34^, the average total radioactivity levels of ^137^Cs in our samples can be fully explained by the presence of only one radioactive particles. Even the highest value of total radioactivity in our sample (4.83 Bq/sample) can be explained by only a few highly radioactive particles. Therefore, our findings suggest that the presence of highly radioactive particles may have caused the observed sporadic elevation of ^137^Cs concentration in plankton. In addition, the discrepancy of the ^137^Cs/^133^Cs ratios in seawater and zooplankton also could be accounted for by the existence of the particles, in which the ^133^Cs content may have been much less than that in seawater.

## Concluding Remarks

The sporadic rises in ^137^Cs concentration and the discrepancy of the ^137^Cs/^133^Cs ratios in seawater and zooplankton could not be explained fully by the uptake of dissolved cesium isotopes from seawater, the incorporation of resuspended bottom sediment onto/into zooplankton, or variability in the taxonomic composition. The presence of insoluble particles enriched in ^137^Cs is the only explanation for these observations. However, concentrations of cesium isotopes in the particles have not been evaluated. Furthermore, whether the particles existed inside the plankton body or adhered to the surface and how the particles can be transferred to predators feeding on the zooplankton also remains to be clarified. Further investigations of the particles with respect to their chemical, physical, and biological characteristics are required to better understand the behavior of radiocesium in the marine food web.

## Methods

### Sample collection

Zooplankton samples were obtained in the daytime by towing a net (160-cm mouth diameter, 0.5-mm mesh) horizontally for 30 min at a depth of around 50 m. The volume of water filtered through the net was measured using a flow meter mounted in the mouth. To ensure sufficient amounts of zooplankton for radionuclide measurements, samples from two or three hauls per station were combined into one sample. A few tens of milliliters of each sample were preserved immediately in 5% (v/v) formalin–seawater buffered with borax for analysis of taxonomic composition, and the remainder was frozen at −20 °C until radiocesium measurement. The collection procedures of seawater and surface sediments have been described previously^21^,^35^.

### Measurements of zooplankton abundance and biomass

Zooplankton species were identified and enumerated under a stereomicroscope. Identification of taxa to the class level mainly followed Chihara and Murano^36^. Abundance and biomass per unit volume of seawater were calculated based on zooplankton counts, sample weight, and volume of water filtered by the plankton net.

### Measurements of radiocesium (^134^Cs and ^137^Cs)

Zooplankton samples were first weighed (wet weight) and then dried in an oven at 105 °C to obtain dry weight. Radioactivity levels of ^134^Cs and ^137^Cs in the dried samples were measured by coaxial type Ge detectors for a few hours. The detection limits of ^134^Cs and ^137^Cs were calculated as three times the fluctuation inherent in the background; they were 0.4 Bq/kg-dry weight for counting times of several tens of thousands of seconds. The concentrations of ^134^Cs and ^137^Cs in the samples were decay-corrected to the sampling date.

The methods used to measure concentrations of ^134^Cs and ^137^Cs in seawater and surface sediments have been described previously^21^,^35^. The data on ^134^Cs and ^137^Cs in seawater and sediments were obtained from webpages maintained by the Secretariat of the Nuclear Regulation Authority^37^,^38^,^39^,^40^ and Takata *et al*.^41^.

### Measurements of stable cesium isotope and aluminum

A part of each dried plankton sample was ground using an agate mortar and pestle and then stored in a glass bottle in the dark until analysis. For elemental analyses, 100 mg of dried zooplankton sample was digested with 7 ml of 15.3 M HNO_3_ in a pressurized microwave digester (Analytik Jena, TOPwave). Concentrations of ^133^Cs and aluminum in this acidic solution were measured by an inductively coupled plasma–mass spectrometer (ICP-MS; Agilent 8800 ICP-QQQ, Agilent Japan); duplicate measurements were performed three times for each sample. Analysis of marine organism reference material (NIST-1566a: oyster tissue), which was processed along with the samples, resulted in good agreement (±10%) for the elements determined.

Concentrations of ^133^Cs in seawater were measured only in the samples collected in November 2014 and January and February 2015 by ICP-MS based on the preconcentration method^42^, which combines adsorption of ^133^Cs on ammonium 12-molybdophosphate (AMP) and an ion exchange resin column. The instrumental detection limit was defined as three standard deviations of the blank solution value (0.0022–0.0030 nmol/L, *n* = 5). We also determined a procedural blank value based on the separation of 20 mL of aqueous AMP solution (*n* = 5), made by adding 3 mg of AMP to a 20-mL aliquot of HNO_3_-acidified Milli-Q water adjusted to a pH of 1.5. This aliquot was then processed in the same manner as the seawater samples. The procedural blank value for our method was about 0.022 nmol/L, which is less than 10% of the concentration range of cesium in coastal waters (about 2.2–3.0 nmol/L^42^). The detection limit for the procedural blank, defined as three times the standard deviation of the blanks, was 0.0075 nmol/L (sample volume, 20 mL). The error was calculated as the standard deviation of three replicate samples measured by ICP-MS.

Concentrations of ^133^Cs in surface sediments were determined only in the samples collected in May 2014 and February 2015 by ICP-MS after complete dissolution by HNO_3_ and HF as described previously^43^. Approximately 10% precision was achieved for the measurements. Concentrations of aluminum in surface sediment samples were measured by inductively coupled plasma–atomic emission spectrometry for the samples collected from May 2012 to January 2014 and ICP-MS for the samples collected from May 2014 to February 2015.

### Autoradiography

To survey possible highly radioactive particles that may have been in the plankton samples, autoradiography was applied to dried zooplankton samples collected at stn. G4 in August 2012 and at stn. J1 in May 2012, which had higher ^137^Cs concentrations than the rest of the samples collected at each station. The samples were spread in plastic zipper bags. An imaging plate (Fujifilm FLA-9000) in contact with the bagged sample was placed in a space shielded with lead blocks and exposed for 24 h for the G4 sample and for 19 h for the J1 sample.

## Additional Information

**How to cite this article**: Ikenoue, T. *et al*. Temporal variation of cesium isotope concentrations and atom ratios in zooplankton in the Pacific off the east coast of Japan. *Sci. Rep.* **7**, 39874; doi: 10.1038/srep39874 (2017).

**Publisher's note:** Springer Nature remains neutral with regard to jurisdictional claims in published maps and institutional affiliations.

## Supplementary Material

Supplementary Information
