# Supplementary material for: Temporal variation of cesium isotope concentrations and atom ratios in zooplankton in the Pacific off the east coast of Japan
Source: Sci Rep. 2017 Jan 4;7:39874. doi: 10.1038/srep39874 (PMC5209667; doi:10.1038/srep39874)
Supplement: Supplementary Information [file srep39874-s1.pdf]

Supplementary information for Temporal variation of cesium isotope concentrations and atom ratios in zooplankton in the Pacific off the east coast of Japan

Takahito Ikenoue[a,*], Hyoe Takata[a], Masashi Kusakabe[a], Natsumi Kudo[a], Kazuyuki Hasegawa[a], Takashi Ishimaru[b, c]

[a] Central Laboratory, Marine Ecology Research Institute, 300 Iwawada, Onjuku-machi, Isumi-gun, Chiba 299-5105, Japan

[b] Marine Ecology Research Institute, Tohwa-Edogawbashi Bldg., 347 Yamabuki-cho, Shinjuku-ku, Tokyo 162-0801, Japan

[c] Department of Marine Science, Tokyo University of Marine Science and Technology, 5-7, Konan 4, Minato-ku, Tokyo 108-8477, Japan

*Corresponding author: Takahito Ikenoue, E-mail: ikenoue@kaiseiken.or.jp

Table S1. Summaries of zooplankton samples, zooplankton abundance, and class-level abundance.

Stn.[a]	Sampling date (JST)	Sampling layer (m)	Sample weight (g-wet)	Flow water volume (m^3)	Biomass (mg-wet/m^3)	Water content (%)	Zooplankton abundance (individual/m^3)	Class-level abundance (individual/m^3)								
								Hydrozoa	Branchiopoda	Maxillopoda	Malacostraca	Sagittoidea	Thaliacea	Appendiculata	Osteichthyes	Others
Cruise: F12OS/1																
B3	29-May-2012	50	230	9578	24	91	26.8	1.9	0.1	11.4	11.0	1.1	0.0	0.1	1.1	0.1
E1	25-May-2012	50	1335	19521	68	91	122.1	29.6	0.0	46.5	43.8	1.4	0.9	0.0	0.0	0.0
E5	26-May-2012	75	1467	23207	63	94	50.2	7.4	0.2	25.5	10.7	2.1	1.9	1.3	0.0	1.1
G0	20-May-2012	50	446	20617	22	88	35.0	1.9	0.1	23.8	7.6	0.9	0.3	0.4	0.0	0.0
G4	21-May-2012	60	1244	22119	56	91	81.4	24.0	0.8	19.5	34.1	0.0	2.6	0.0	0.0	0.4
J1	16-May-2012	20	106	12355	9	93	4.6	0.4	0.1	1.8	0.8	0.0	0.0	0.0	1.5	0.0
J3	18-May-2012	50	772	19973	39	94	53.3	10.3	0.8	10.8	24.7	0.5	3.4	1.3	0.0	1.5
Cruise: F12OS/2																
B3	02-Aug-2012	43	1769	25333	70	90	65.9	6.7	0.0	28.2	18.9	10.5	0.9	0.0	0.2	0.5
E1	06-Aug-2012	60	1067	12901	83	94	58.4	19.3	0.6	28.1	6.9	0.8	1.7	0.3	0.6	0.3
E5	04-Aug-2012	45-65	234	17042	14	95	12.3	2.7	0.5	6.8	0.7	1.2	0.0	0.2	0.1	0.1
G0	08-Aug-2012	50	1506	22211	68	94	37.3	10.5	0.0	17.9	4.9	2.7	0.9	0.0	0.2	0.2
G4	09-Aug-2012	50-70	1682	19130	88	94	45.5	0.6	3.3	27.0	3.9	3.1	2.7	1.8	0.4	2.7
J1	13-Aug-2012	35	1119	10915	103	94	113.4	47.5	0.0	18.8	9.2	27.7	7.9	0.0	0.7	1.7
J3	12-Aug-2012	70	706	17370	41	94	31.0	11.2	0.9	11.1	2.3	2.3	1.4	0.4	1.1	0.3
Cruise: F12OS/3																
B3	31-Oct-2012	50	1560	20661	76	92	47.8	3.9	6.7	25.5	3.0	7.6	0.0	0.3	0.0	0.8
E1	27-Oct-2012	50	1137	23417	49	92	50.5	5.3	3.2	28.6	4.9	6.0	0.2	0.8	0.6	0.8
E5	26-Oct-2012	50	1759	22996	77	93	28.9	3.3	0.0	17.0	0.8	3.2	1.1	2.2	0.3	1.1
G0	24-Oct-2012	50	892	26238	34	91	20.9	0.5	0.0	11.9	3.1	4.2	0.3	0.8	0.1	0.0
G4	04-Nov-2012	50	1727	21967	79	93	90.9	6.3	0.0	55.2	4.5	19.6	1.0	2.1	0.0	2.1
J1	07-Nov-2012	50	1637	18294	89	89	161.6	1.2	2.4	112.1	22.7	20.9	0.0	0.0	0.6	1.8
Cruise: F12OS/4																
B3	16-Jan-2013	50	685	21913	31	94	40.6	4.4	0.7	22.5	1.8	2.8	0.3	6.0	0.1	1.9
E1	19-Jan-2013	50	799	20228	39	94	23.3	1.5	0.0	9.9	3.6	2.3	0.6	4.7	0.1	0.6
E5	24-Jan-2013	50	213	20561	10	92	16.9	4.4	0.0	7.8	2.5	1.7	0.1	0.1	0.1	0.1
G0	18-Jan-2013	50	298	25547	12	92	12.2	1.0	0.0	6.9	1.8	1.8	0.2	0.3	0.0	0.1
G4	25-Jan-2013	50	296	44986	7	92	8.2	2.3	0.0	1.9	3.2	0.4	0.1	0.2	0.0	0.1
J1	12-Jan-2013	30	1114	11322	98	95	51.6	1.7	0.9	12.5	2.2	4.4	0.9	27.8	0.7	0.7
J3	12-Jan-2013	80	455	24966	18	94	15.6	1.4	0.0	7.8	2.6	1.0	0.2	2.3	0.1	0.2
Cruise: F13OS/1																
B3	31-May-2013	50	747	22618	33.0	91	47.1	0.0	0.4	40.5	1.3	4.8	0.0	0.0	0.0	0.0
E1	27-May-2013	50	1648	26020	63.3	90	60.0	0.0	0.0	53.2	6.8	0.0	0.0	0.0	0.0	0.0
E5	26-May-2013	50	5298	9414	562.8	89	465.2	0.0	0.0	397.7	22.5	22.5	0.0	0.0	0.0	22.5
G0	25-May-2013	50	1290	21365	60.4	89	128.0	0.0	0.0	126.4	1.6	0.0	0.0	0.0	0.0	0.0
G4	22-May-2013	50	2121	21265	99.7	92	210.1	6.6	0.0	170.2	14.6	0.0	0.0	9.3	9.3	0.0
I1	20-May-2013	50	1816	23081	78.7	88	70.3	0.0	0.0	65.0	5.2	0.0	0.0	0.0	0.0	0.0
J1	19-May-2013	30	1431	23532	60.8	92	39.9	4.4	0.0	29.7	3.4	1.9	0.0	0.0	0.5	0.0
J3	18-May-2013	50	2155	23548	91.5	90	123.2	0.0	0.0	118.3	2.4	2.4	0.0	0.0	0.0	0.0
Cruise: F13OS/2																
B3	24-Aug-2013	50	1291	13234	97.6	94	70.9	0.0	25.4	13.0	1.3	7.8	19.5	0.0	2.6	1.3
E1	21-Aug-2013	50	971	17470	55.6	94	41.1	1.5	4.1	6.3	1.1	12.6	13.7	0.7	0.4	0.7
E5	22-Aug-2013	50	616	17539	35.1	89	18.3	0.0	0.0	16.6	0.6	0.8	0.0	0.0	0.0	0.3
G0	20-Aug-2013	50	593	20757	28.6	91	23.3	0.2	6.1	5.9	0.8	6.1	3.8	0.1	0.3	0.0
G4	17-Aug-2013	50	525	19546	26.9	90	20.1	0.0	0.0	14.4	3.7	1.5	0.3	0.0	0.0	0.2
I1	15-Aug-2013	50	877	21129	41.5	92	75.8	3.3	0.6	33.7	2.8	26.0	3.3	0.6	3.9	1.7
J1	14-Aug-2013	30	3063	13701	223.5	88	152.0	0.0	0.0	122.2	20.9	8.9	0.0	0.0	0.0	0.0
J3	12-Aug-2013	50	1507	17347	86.9	93	147.1	3.5	83.4	48.6	1.2	5.8	0.0	0.0	1.2	3.5

Table S1 (continued)

Stn.[a]	Sampling date (JST)	Sampling layer (m)	Sample weight (g-wet)	Flow water volume (m^3)	Biomass (mg-wet/m^3)	Water content (%)	Zooplankton abundance (individual/m^3)	Class-level abundance (individual/m^3)								
								Hydrozoa	Branchiopoda	Maxillopoda	Malacostraca	Sagittoidea	Thaliacea	Appendiculata	Osteichthyes	Others
Cruise: F13OS/3																
B3	05-Nov-2013	50	400	17556	22.8	94	19.9	1.0	0.5	11.2	1.0	5.3	0.2	0.7	0.0	0.0
E1	09-Nov-2013	50	1423	14922	95.4	91	103.0	7.6	2.5	62.3	2.5	21.6	1.3	3.8	0.0	1.3
E5	14-Nov-2013	50	637	19424	32.8	92	37.2	2.6	0.0	24.1	1.7	3.5	3.5	1.7	0.0	0.0
G0	19-Nov-2013	50	982	10502	93.5	92	145.8	12.5	1.2	99.7	5.0	17.5	5.0	1.2	0.0	3.7
G4	23-Nov-2013	50	700	17722	39.5	95	42.1	3.2	3.2	24.7	1.1	6.3	3.7	0.0	0.0	0.0
I1	11-Nov-2013	50	616	18163	33.9	92	56.2	2.0	0.0	32.6	3.3	12.6	0.0	4.9	0.0	0.8
J1	17-Nov-2013	50	1694	17329	97.7	92	81.3	4.2	4.2	46.9	17.7	5.2	2.1	0.0	0.0	1.0
J3	21-Nov-2013	50	771	16662	46.3	91	64.2	3.1	4.9	40.1	5.6	4.9	1.9	3.1	0.0	0.6
Cruise: F13OS/4																
B3	27-Jan-2014	50	541	20355	26.6	94	12.4	1.6	0.0	8.0	0.4	1.8	0.0	0.7	0.0	0.0
E1	23-Jan-2014	50	164	31760	5.2	93	6.3	0.3	0.0	3.9	0.3	1.2	0.0	0.5	0.1	0.0
E5	24-Jan-2014	50	65	27989	2.3	95	0.3	0.1	0.0	0.2	0.0	0.0	0.0	0.0	0.0	0.0
G0	18-Jan-2014	50	220	19955	11.0	91	8.1	0.4	0.0	5.9	0.1	0.7	0.0	0.6	0.4	0.1
G4	17-Jan-2014	150	144	39176	3.7	94	1.0	0.0	0.0	0.7	0.0	0.1	0.0	0.0	0.0	0.2
I1	15-Jan-2014	50	160	22781	7.0	92	4.7	0.4	0.0	3.5	0.2	0.1	0.0	0.4	0.1	0.0
J1	13-Jan-2014	30	313	15292	20.4	93	11.2	0.0	0.0	7.9	1.9	0.8	0.0	0.0	0.5	0.0
J3	14-Jan-2014	50	32	28428	1.1	82	0.4	0.0	0.0	0.2	0.0	0.1	0.0	0.0	0.0	0.0
Cruise: F14OS/1																
B3	14-May-2014	50	2247	18830	119.3	88	259.3	8.0	0.0	225.9	14.3	1.6	3.2	6.4	0.0	0.0
E1	17-May-2014	50	2041	22859	89.3	88	317.9	4.8	0.0	296.5	9.5	0.0	1.2	2.4	1.2	2.4
E5	19-May-2014	50	2566	26475	96.9	90	119.8	0.0	0.0	115.2	1.2	2.3	0.0	0.0	0.0	1.2
G0	24-May-2014	50	1907	26067	73.1	91	147.5	3.5	0.0	134.3	0.9	0.0	0.0	6.1	0.9	1.8
J1	27-May-2014	30	2152	22089	97.4	87	227.6	4.2	0.0	174.6	35.3	4.2	0.0	0.0	1.0	8.3
Cruise: F14OS/2																
B3	17-Aug-2014	50	1038	20137	51.6	90	96.1	0.0	2.5	59.0	2.9	27.2	2.5	1.2	0.0	0.8
E1	22-Aug-2014	50	1068	20343	52.5	92	66.6	0.6	1.7	29.7	4.5	10.1	17.9	1.1	0.6	0.6
E5	20-Aug-2014	50	1230	17784	69.2	93	44.5	0.0	0.0	29.1	4.7	4.4	5.0	0.0	0.0	1.4
G0	31-Aug-2014	50	872	22416	38.9	88	58.1	0.0	2.5	25.7	0.4	23.2	1.2	0.0	0.8	4.2
J1	30-Aug-2014	30	1961	17656	111.1	88	349.3	2.7	92.0	145.3	26.7	50.7	18.7	0.0	13.3	0.0
Cruise: F14OS/3																
B3	04-Nov-2014	50	572	16985	33.7	91	41.2	2.5	3.8	21.1	4.4	8.2	0.3	0.3	0.0	0.6
E1	17-Nov-2014	50	1561	15533	100.5	93	151.4	6.7	2.7	64.3	6.7	29.5	2.7	34.8	0.0	4.0
E5	05-Nov-2014	50	1351	15559	86.8	94	141.7	5.6	0.0	75.0	5.6	31.9	1.4	11.1	1.4	9.7
G0	18-Nov-2014	50	1165	16395	71.0	92	104.6	3.8	0.8	65.9	3.0	3.8	1.5	20.5	0.8	4.5
J1	14-Nov-2014	30	1054	12254	86.0	93	75.0	6.9	0.0	45.4	2.8	8.3	0.7	5.5	0.7	4.8
Cruise: F14OS/4																
B3	03-Feb-2015	50	252	23741	10.6	88	9.9	0.2	0.0	9.2	0.1	0.2	0.0	0.0	0.1	0.3
E1	06-Feb-2015	50	285	25086	11.4	92	8.1	0.2	0.0	6.5	0.3	0.3	0.0	0.5	0.0	0.4
E5	29-Jan-2015	50	210	28381	7.4	94	4.3	0.2	0.0	3.3	0.2	0.1	0.0	0.0	0.0	0.6
G0	26-Jan-2015	50	79	19969	4.0	90	7.7	0.1	0.2	6.9	0.1	0.3	0.0	0.0	0.0	0.1
J1	19-Jan-2015	30	28	24050	1.2	91	0.9	0.1	0.0	0.4	0.3	0.1	0.0	0.0	0.0	0.0

a: See Oikawa et al.[35] and Fig. 1 for the sampling locations.

Table S2. Concentrations of ^{134}Cs and ^{137}Cs in zooplankton samples and in seawater samples collected off the east coast of Japan.

Stn.	Zooplankton					Seawater		
	^{134}Cs (Bq/kg-dry)	^{137}Cs (Bq/kg-dry)	^{134}Cs (Bq/kg-wet)	^{137}Cs (Bq/kg-wet)	aCR of ^{137}Cs (L/kg-wet)	Sampling depth (m)	^{134}Cs (mBq/L)	^{137}Cs (mBq/L)
Cruise: F13OS/1 (May 2013)								
B3	ND	0.82 ± 0.14	ND	0.07 ± 0.01	49	50	ND	1.5 ± 0.2
E1	ND	0.26 ± 0.08	ND	0.03 ± 0.01	13	50	ND	2.0 ± 0.2
E5	ND	0.28 ± 0.07	ND	0.03 ± 0.01	20	50	ND	1.5 ± 0.2
G0	0.92 ± 0.12	1.99 ± 0.13	0.10 ± 0.01	0.22 ± 0.01	136	50	ND	1.6 ± 0.2
G4	LTD	0.36 ± 0.08	LTD	0.03 ± 0.01	13	50	ND	2.3 ± 0.2
I1	0.61 ± 0.07	1.03 ± 0.08	0.07 ± 0.01	0.12 ± 0.01	69	50	ND	1.8 ± 0.2
J1	1.51 ± 0.14	2.59 ± 0.15	0.12 ± 0.01	0.20 ± 0.01	26	37	3.2 ± 0.2	7.8 ± 0.3
J3	ND	0.45 ± 0.07	ND	0.04 ± 0.01	23	50	0.5 ± 0.2	1.9 ± 0.2
Cruise: F13OS/2 (Aug. 2013)								
B3	0.65 ± 0.13	1.73 ± 0.14	0.04 ± 0.01	0.11 ± 0.01	56	50	ND	1.9 ± 0.2
E1	1.52 ± 0.22	3.12 ± 0.22	0.09 ± 0.01	0.18 ± 0.01	128	50	ND	1.4 ± 0.2
E5	0.46 ± 0.15	0.95 ± 0.14	0.05 ± 0.02	0.11 ± 0.02	66	50	ND	1.6 ± 0.2
G0	1.45 ± 0.26	3.09 ± 0.24	0.12 ± 0.02	0.26 ± 0.02	68	50	1.1 ± 0.2	3.9 ± 0.2
G4	0.97 ± 0.19	1.72 ± 0.18	0.09 ± 0.02	0.17 ± 0.02	104	50	ND	1.6 ± 0.2
I1	3.94 ± 0.18	8.79 ± 0.20	0.32 ± 0.01	0.72 ± 0.02	481	50	ND	1.5 ± 0.2
J1	1.97 ± 0.10	4.12 ± 0.11	0.23 ± 0.01	0.48 ± 0.01	66	38	2.8 ± 0.3	7.3 ± 0.3
J3	0.50 ± 0.13	0.73 ± 0.12	0.03 ± 0.01	0.05 ± 0.01	18	50	ND	2.7 ± 0.2
Cruise: F13OS/3 (Nov. 2013)								
B3	ND	0.65 ± 0.11	ND	0.04 ± 0.01	19	50	ND	2.0 ± 0.1
E1	0.72 ± 0.22	2.23 ± 0.26	0.07 ± 0.02	0.21 ± 0.02	80	50	0.7 ± 0.2	2.6 ± 0.2
E5	ND	2.57 ± 0.44	ND	0.20 ± 0.03	93	50	ND	2.1 ± 0.2
G0	3.50 ± 0.35	7.25 ± 0.39	0.29 ± 0.03	0.61 ± 0.03	264	50	ND	2.3 ± 0.2
G4	3.44 ± 0.53	6.94 ± 0.58	0.19 ± 0.03	0.38 ± 0.03	159	200	ND	2.4 ± 0.2
I1	ND	ND ±	ND	ND	—	50	ND	3.0 ± 0.2
J1	ND	0.78 ± 0.22	ND	0.06 ± 0.02	23	39	ND	2.6 ± 0.2
J3	ND	1.18 ± 0.32	ND	0.10 ± 0.03	31	50	0.8 ± 0.2	3.4 ± 0.2
Cruise: F13OS/4 (Jan. 2014)								
B3	8.34 ± 0.70	20.2 ± 0.9	0.49 ± 0.04	1.18 ± 0.05	392	50	ND	3.0 ± 0.2
E1	3.64 ± 0.71	6.62 ± 0.72	0.26 ± 0.05	0.48 ± 0.05	251	50	ND	1.9 ± 0.2
E5	12.9 ± 1.1	33.3 ± 1.1	0.68 ± 0.06	1.76 ± 0.06	801	50	ND	2.2 ± 0.2
G0	5.14 ± 0.90	12.4 ± 0.9	0.47 ± 0.08	1.12 ± 0.08	163	50	1.9 ± 0.2	6.9 ± 0.2
G4	ND	6.59 ± 1.63	ND	0.38 ± 0.09	189	50	ND	2.0 ± 0.2
I1	LTD	9.00 ± 0.70	LTD	0.70 ± 0.05	54	50	5.0 ± 0.3	13.0 ± 0.4
J1	4.47 ± 0.70	8.65 ± 0.74	0.31 ± 0.05	0.60 ± 0.05	74	40	2.6 ± 0.3	8.2 ± 0.3
J3	17.1 ± 1.9	40.3 ± 1.9	3.14 ± 0.36	7.39 ± 0.34	2957	100	ND	2.5 ± 0.2

Table S2 (continued)

Stn.	Zooplankton					Seawater		
	^{134}Cs (Bq/kg-dry)	^{137}Cs (Bq/kg-dry)	^{134}Cs (Bq/kg-wet)	^{137}Cs (Bq/kg-wet)	aCR of ^{137}Cs (L/kg-wet)	Sampling depth (m)	^{134}Cs (mBq/L)	^{137}Cs (mBq/L)
Cruise: F14OS/1 (May 2014)								
B3	0.74 ± 0.15	2.06 ± 0.19	0.09 ± 0.02	0.24 ± 0.02	45	50	1.7 ± 0.3	5.4 ± 0.2
E1	7.90 ± 0.26	19.9 ± 0.4	0.95 ± 0.03	2.38 ± 0.05	661	50	1.0 ± 0.3	3.6 ± 0.2
E5	1.07 ± 0.15	2.96 ± 0.19	0.11 ± 0.02	0.30 ± 0.02	213	50	ND	1.4 ± 0.2
G0	ND	1.20 ± 0.19	ND	0.11 ± 0.02	39	50	ND	2.8 ± 0.2
J1	1.20 ± 0.16	3.38 ± 0.21	0.15 ± 0.02	0.43 ± 0.03	96	39	1.5 ± 0.2	4.5 ± 0.2
Cruise: F14OS/2 (Aug. 2014)								
B3	LTD	2.35 ± 0.19	LTD	0.24 ± 0.02	141	50	ND	1.7 ± 0.2
E1	0.43 ± 0.12	0.58 ± 0.13	0.04 ± 0.01	0.05 ± 0.01	25	50	ND	1.9 ± 0.2
E5	ND	LTD	ND	LTD	—	50	ND	1.9 ± 0.2
G0	0.46 ± 0.14	1.64 ± 0.15	0.05 ± 0.02	0.19 ± 0.02	90	50	ND	2.1 ± 0.2
J1	0.59 ± 0.09	1.54 ± 0.10	0.07 ± 0.01	0.18 ± 0.01	77	40	ND	2.4 ± 0.2
Cruise: F14OS/3 (Nov. 2014)								
B3	1.15 ± 0.17	3.88 ± 0.19	0.10 ± 0.01	0.34 ± 0.02	188	50	ND	1.8 ± 0.2
E1	1.28 ± 0.15	3.01 ± 0.15	0.09 ± 0.01	0.21 ± 0.01	97	50	ND	2.2 ± 0.2
E5	1.45 ± 0.18	3.54 ± 0.15	0.09 ± 0.01	0.22 ± 0.01	96	50	ND	2.3 ± 0.2
G0	0.86 ± 0.11	2.80 ± 0.12	0.07 ± 0.01	0.23 ± 0.01	92	50	ND	2.5 ± 0.2
J1	0.74 ± 0.17	2.26 ± 0.18	0.05 ± 0.01	0.16 ± 0.01	68	33	ND	2.3 ± 0.2
Cruise: F14OS/4 (Jan.-Feb. 2015)								
B3	ND	2.61 ± 0.43	ND	0.31 ± 0.05	157	50	ND	2.0 ± 0.2
E1	LTD	3.38 ± 0.43	LTD	0.26 ± 0.03	103	50	ND	2.5 ± 0.2
E5	2.27 ± 0.49	2.29 ± 0.49	0.14 ± 0.03	0.15 ± 0.03	76	50	ND	1.9 ± 0.2
G0	2.78 ± 0.92	7.29 ± 0.66	0.29 ± 0.09	0.75 ± 0.07	140	50	1.5 ± 0.2	5.4 ± 0.3
J1	ND	13.8 ± 1.5	ND	1.26 ± 0.14	350	37	ND	3.6 ± 0.2

Underlined numbers indicate the samples containing a portion of phytoplankton.
ND: not detected or below the detection limit.
LTD: below 3σ of the counting statistics.

Table S3. Concentrations of ^{133}Cs and aluminum (Al) and ^{137}Cs/^{133}Cs ratios in zooplankton samples and concentrations of ^{133}Cs and ^{137}Cs/^{133}Cs ratios in seawater samples.

Stn.	Zooplankton			Seawater	
	^{133}Cs (ng/g-dry)	Al (μg/g-dry)	^{137}Cs/^{133}Cs ($\times 10^{-7}$)	^{133}Cs (ng/L)	^{137}Cs/^{133}Cs ($\times 10^{-9}$)
Cruise: F12OS/1 (May 2012)					
B3	54 ± 10	336 ± 36	0.18 ± 0.06	—	5.0 ± 0.3
E1	31 ± 3	23 ± 8	0.17 ± 0.04	—	5.9 ± 0.3
E5	31 ± 1	40 ± 5	0.17 ± 0.04	—	5.3 ± 0.3
G0	46 ± 6	201 ± 18	1.47 ± 0.21	—	18.7 ± 1.1
G4	39 ± 2	94 ± 13	0.48 ± 0.05	—	7.4 ± 0.4
J1	140 ± 6	1667 ± 301	4.11 ± 0.21	—	16.8 ± 1.0
J3	61 ± 4	346 ± 13	1.09 ± 0.08	—	8.5 ± 0.5
Cruise: F12OS/2 (Aug. 2012)					
B3	37 ± 4	39 ± 4	0.10 ± 0.02	—	1.9 ± 0.1
E1	87 ± 22	42 ± 2	—	—	2.8 ± 0.2
E5	62 ± 11	208 ± 20	—	—	3.4 ± 0.2
G0	39 ± 11	42 ± 14	0.26 ± 0.08	—	4.3 ± 0.3
G4	54 ± 15	345 ± 55	0.94 ± 0.27	—	4.0 ± 0.2
J1	65 ± 34	62 ± 11	0.46 ± 0.24	—	13.8 ± 0.8
J3	40 ± 7	72 ± 22	0.24 ± 0.06	—	4.0 ± 0.2
Cruise: F12OS/3 (Oct.-Nov. 2012)					
B3	46 ± 11	64 ± 23	0.10 ± 0.03	—	3.2 ± 0.2
E1	42 ± 4	122 ± 31	0.46 ± 0.06	—	5.0 ± 0.3
E5	47 ± 32	45 ± 5	—	—	2.7 ± 0.2
G0	32 ± 3	62 ± 16	1.27 ± 0.14	—	3.7 ± 0.2
G4	34 ± 2	38 ± 6	0.45 ± 0.04	—	2.2 ± 0.1
J1	92 ± 3	810 ± 42	0.49 ± 0.02	—	5.2 ± 0.4
Cruise: F12OS/4 (Jan. 2013)					
B3	379 ± 50	4996 ± 464	0.25 ± 0.03	—	3.2 ± 0.2
E1	60 ± 7	386 ± 23	3.00 ± 0.34	—	2.6 ± 0.1
E5	53 ± 1	234 ± 12	0.17 ± 0.05	—	2.6 ± 0.1
G0	92 ± 7	706 ± 68	0.30 ± 0.04	—	3.1 ± 0.2
G4	66 ± 10	382 ± 42	0.12 ± 0.04	—	2.8 ± 0.2
J1	174 ± 8	1986 ± 123	0.91 ± 0.05	—	4.1 ± 0.3
J3	77 ± 5	751 ± 36	0.24 ± 0.03	—	3.7 ± 0.3
Cruise: F13OS/1 (May 2013)					
B3	47 ± 3	107 ± 19	0.05 ± 0.01	—	1.5 ± 0.2
E1	37 ± 8	18 ± 4	0.02 ± 0.01	—	2.0 ± 0.2
E5	32 ± 3	18 ± 6	0.03 ± 0.01	—	1.5 ± 0.2
G0	37 ± 1	75 ± 83	0.17 ± 0.01	—	1.6 ± 0.2
G4	32 ± 5	103 ± 113	0.03 ± 0.01	—	2.3 ± 0.2
I1	60 ± 36	51 ± 3	0.05 ± 0.03	—	1.8 ± 0.2
J1	79 ± 30	91 ± 9	0.10 ± 0.04	—	7.7 ± 0.3
J3	47 ± 20	72 ± 25	0.03 ± 0.01	—	1.9 ± 0.2
Cruise: F13OS/2 (Aug. 2013)					
B3	55 ± 14	172 ± 13	0.10 ± 0.03	—	1.9 ± 0.2
E1	71 ± 25	158 ± 5	0.14 ± 0.05	—	1.4 ± 0.2
E5	46 ± 14	126 ± 9	0.07 ± 0.02	—	1.6 ± 0.2
G0	67 ± 12	145 ± 5	0.14 ± 0.03	—	3.8 ± 0.2
G4	47 ± 5	140 ± 7	0.11 ± 0.02	—	1.6 ± 0.2
I1	88 ± 18	260 ± 4	0.31 ± 0.06	—	1.5 ± 0.2
J1	73 ± 20	139 ± 11	0.18 ± 0.05	—	7.2 ± 0.3
J3	46 ± 2	133 ± 21	0.05 ± 0.01	—	2.7 ± 0.2

Table S3 (continued)

Stn.	Zooplankton ^{133}Cs (ng/g-dry)	Zooplankton Al (μg/g-dry)	Zooplankton $^{137}Cs/^{133}Cs$ ($\times 10^{-7}$)	Seawater ^{133}Cs (ng/L)	Seawater $^{137}Cs/^{133}Cs$ ($\times 10^{-9}$)
Cruise: F13OS/3 (Nov. 2013)					
B3	53 ± 5	125 ± 6	0.04 ± 0.01	—	2.0 ± 0.1
E1	51 ± 3	87 ± 4	0.14 ± 0.02	—	2.6 ± 0.2
E5	44 ± 4	138 ± 13	0.18 ± 0.03	—	2.1 ± 0.2
G0	54 ± 5	147 ± 11	0.42 ± 0.04	—	2.3 ± 0.2
G4	111 ± 70	512 ± 10	0.19 ± 0.12	—	2.4 ± 0.2
I1	90 ± 45	111 ± 12	—	—	3.0 ± 0.2
J1	72 ± 27	152 ± 8	0.03 ± 0.02	—	2.6 ± 0.2
J3	80 ± 29	291 ± 13	0.05 ± 0.02	—	3.4 ± 0.2
Cruise: F13OS/4 (Jan. 2014)					
B3	261 ± 24	3042 ± 123	0.24 ± 0.02	—	3.0 ± 0.2
E1	76 ± 13	353 ± 22	0.27 ± 0.05	—	1.9 ± 0.2
E5	214 ± 7	2209 ± 27	0.49 ± 0.02	—	2.2 ± 0.2
G0	73 ± 8	326 ± 11	0.53 ± 0.07	—	6.8 ± 0.3
G4	153 ± 29	854 ± 21	0.13 ± 0.04	—	2.0 ± 0.2
I1	109 ± 7	400 ± 15	0.26 ± 0.03	—	12.8 ± 0.5
J1	70 ± 3	342 ± 14	0.39 ± 0.04	—	8.1 ± 0.3
J3	259 ± 13	2452 ± 23	0.49 ± 0.03	—	2.5 ± 0.2
Cruise: F14OS/1 (May 2014)					
B3	52 ± 9	81 ± 2	0.12 ± 0.02	—	5.3 ± 0.3
E1	46 ± 1	42 ± 3	1.36 ± 0.04	—	3.6 ± 0.2
E5	34 ± 3	38 ± 2	0.27 ± 0.03	—	1.4 ± 0.2
G0	28 ± 25	75 ± 92	0.13 ± 0.12	—	2.8 ± 0.2
J1	60 ± 10	135 ± 6	0.18 ± 0.03	—	4.4 ± 0.2
Cruise: F14OS/2 (Aug. 2014)					
B3	55 ± 2	119 ± 7	0.13 ± 0.01	—	1.7 ± 0.2
E1	41 ± 4	90 ± 3	0.04 ± 0.01	—	1.9 ± 0.2
E5	39 ± 3	41 ± 7	—	—	1.9 ± 0.2
G0	44 ± 1	91 ± 4	0.12 ± 0.01	—	2.1 ± 0.2
J1	41 ± 1	57 ± 1	0.12 ± 0.01	—	2.4 ± 0.2
Cruise: F14OS/3 (Nov. 2014)					
B3	42 ± 5	244 ± 8	0.29 ± 0.04	325 ± 2	1.8 ± 0.2
E1	36 ± 4	113 ± 24	0.26 ± 0.04	329 ± 24	2.2 ± 0.2
E5	36 ± 3	206 ± 17	0.31 ± 0.03	315 ± 5	2.3 ± 0.2
G0	29 ± 1	70 ± 10	0.30 ± 0.02	310 ± 1	2.5 ± 0.2
J1	26 ± 2	133 ± 4	0.27 ± 0.03	315 ± 11	2.3 ± 0.2
Cruise: F14OS/4 (Jan.-Feb. 2015)					
B3	147 ± 7	2241 ± 393	0.06 ± 0.01	319 ± 10	2.0 ± 0.2
E1	104 ± 6	1657 ± 151	0.10 ± 0.01	310 ± 1	2.5 ± 0.2
E5	28 ± 13	193 ± 28	0.26 ± 0.13	307 ± 0.3	1.9 ± 0.2
G0	67 ± 3	365 ± 17	0.34 ± 0.04	315 ± 6	5.3 ± 0.3
J1	143 ± 21	1424 ± 94	0.30 ± 0.06	317 ± 6	3.6 ± 0.2

Table S4. Comparison of ^{133}Cs concentrations in zooplankton collected in coastal and offshore waters near Japan.

Concentration (ng/g-dry)	Sampling date	Locations	References
73	Sep. 1984	Japan Sea	Masuzawa et al.[10]
12 to 447 (average±SD: 77±97)	23 June1992 to 8 Feb. 1994	off the Misaki coast (Kanagawa pref.)	Tateda[11]
16 to 189 (average±SD: 71±47)	22 July 2011 to 15 Nov. 2013	Sendai Bay (Miyagi pref.)	Kaeriyama et al.[6]
27 to 154 (average±SD: 58±43)	19 Apr. 2012 to 7 Aug. 2012	Joban-Sanriku coast (Fukushima and nearby prefecture)	Kaeriyama et al.[6]
26 to 379 (average±SD: 72±59)	16 May 2012 to 6 Feb. 2015	offshore of Fukushima and nearby prefectures	This study

Table S5. Concentration of ^{137}Cs, ^{133}Cs, and aluminum (Al) in surface sediments and assumed contribution of resuspended sediments to ^{137}Cs and ^{133}Cs in zooplankton.

Stn.	^{137}Cs (Bq/kg-dry)	^{133}Cs (μg/g-dry)	Al (mg/g-dry)	^{137}Cs from sediments (Bq/kg-dry)	^{137}Cs from sediments (%)	^{133}Cs from sediments (ng/g-dry)	^{133}Cs from sediments (%)
Cruise: F12OS/1 (May 2012)							
B3	220 ± 1.0	—	63.8	1.2 ± 0.1	37.3	15 ± 3	27.3
E1	140 ± 0.7	—	63.3	0.051 ± 0.017	3.0	0.92 ± 0.39	3.0
E5	9 ± 0.2	—	64.3	0.0058 ± 0.0007	0.3	2.5 ± 0.5	8.2
G0	250 ± 1.0	—	66.5	0.76 ± 0.07	3.5	9.1 ± 1.5	19.8
G4	56 ± 0.5	—	64.6	0.082 ± 0.011	1.4	5.1 ± 1.6	13.1
J1	37 ± 0.3	—	55.6	1.1 ± 0.2	0.6	39 ± 7	27.6
J3	54 ± 0.5	—	60.6	0.31 ± 0.01	1.5	19 ± 2	31.5
Cruise: F12OS/2 (Aug. 2012)							
B3	220 ± 0.9	—	71.5	0.12 ± 0.01	9.8	1.7 ± 0.3	4.6
E1	240 ± 0.9	—	66.9	—	—	1.7 ± 0.5	1.9
E5	8 ± 0.2	—	69.9	—	—	13 ± 2	21.2
G0	190 ± 0.7	—	68.4	0.12 ± 0.04	3.6	1.9 ± 0.7	4.9
G4	34 ± 0.4	—	67.4	0.17 ± 0.03	1.1	19 ± 6	34.6
J1	46 ± 0.3	—	41.6	0.068 ± 0.012	0.7	1.4 ± 0.3	2.2
J3	32 ± 0.4	—	62.8	0.037 ± 0.011	1.2	4.0 ± 1.2	10.0
Cruise: F12OS/3 (Oct.-Nov. 2012)							
B3	200 ± 0.8	—	74.6	0.17 ± 0.06	12.2	2.8 ± 1.1	6.1
E1	110 ± 0.6	—	70.4	0.19 ± 0.05	3.1	4.9 ± 1.8	11.6
E5	9 ± 0.2	—	73.1	—	—	2.8 ± 0.5	6.0
G0	170 ± 0.8	—	72.6	0.15 ± 0.04	1.1	2.8 ± 0.8	8.8
G4	49 ± 0.4	—	69.5	0.027 ± 0.004	0.6	2.1 ± 0.7	6.1
J1	23 ± 0.2	—	30.1	0.62 ± 0.03	4.2	19 ± 2	20.4
Cruise: F12OS/4 (Jan. 2013)							
B3	197 ± 0.8	—	69.8	14 ± 1	46.8	219 ± 40	57.8
E1	95 ± 0.6	—	65.2	0.56 ± 0.03	1.0	15 ± 4	25.5
E5	6 ± 0.2	—	67.2	0.022 ± 0.001	0.8	15 ± 2	27.9
G0	100 ± 0.6	—	68.3	1.0 ± 0.1	11.9	32 ± 5	34.8
G4	11 ± 0.2	—	67.1	0.063 ± 0.007	2.4	21 ± 6	31.4
J1	19 ± 0.2	—	25.5	1.5 ± 0.1	3.0	46 ± 4	26.4
J3	43 ± 0.4	—	60.3	0.53 ± 0.03	9.0	42 ± 4	54.1
Cruise: F13OS/1 (May 2013)							
B3	100 ± 0.6	—	71.0	0.15 ± 0.03	18.4	4.7 ± 1.1	10.0
E1	100 ± 0.6	—	67.3	0.026 ± 0.006	10.3	0.70 ± 0.24	1.9
E5	5 ± 0.1	—	74.1	0.0011 ± 0.0004	0.4	1.2 ± 0.4	3.6
G0	150 ± 0.8	—	67.9	0.17 ± 0.18	8.4	3.4 ± 3.8	9.2
G4	25 ± 0.3	—	65.1	0.040 ± 0.043	11.0	5.6 ± 6.3	17.5
I1	110 ± 0.7	—	72.3	0.077 ± 0.005	7.5	2.5 ± 0.4	4.2
J1	27 ± 0.3	—	53.0	0.046 ± 0.004	1.8	2.1 ± 0.2	2.7
J3	25 ± 0.4	—	59.6	0.030 ± 0.010	6.7	4.0 ± 1.4	8.5
Cruise: F13OS/2 (Aug. 2013)							
B3	120 ± 0.6	—	72.4	0.29 ± 0.02	16.5	7.5 ± 1.3	13.7
E1	89 ± 0.5	—	66.5	0.21 ± 0.01	6.8	6.3 ± 1.7	8.9
E5	4 ± 0.2	—	70.0	0.0063 ± 0.0005	0.7	7.9 ± 1.2	17.2
G0	110 ± 0.7	—	70.0	0.23 ± 0.01	7.4	6.6 ± 0.9	9.8
G4	39 ± 0.4	—	66.4	0.082 ± 0.004	4.8	7.6 ± 2.1	16.1
I1	200 ± 1.0	—	71.4	0.73 ± 0.01	8.3	13 ± 2	14.5
J1	19 ± 0.3	—	30.6	0.086 ± 0.007	2.1	3.2 ± 0.3	4.4
J3	23 ± 0.4	—	61.9	0.049 ± 0.008	6.8	7.4 ± 1.3	16.0

Table S5 (continued)

Stn.	^{137}Cs (Bq/kg-dry)	^{133}Cs (μg/g-dry)	Al (mg/g-dry)	^{137}Cs from sediments (Bq/kg-dry)	(%)	^{133}Cs from sediments (ng/g-dry)	(%)
Cruise: F13OS/3 (Nov. 2013)							
B3	74 ± 0.5	—	67.8	0.14 ± 0.01	20.9	5.5 ± 0.9	10.4
E1	42 ± 0.4	—	66.8	0.055 ± 0.003	2.4	3.4 ± 1.0	6.8
E5	5 ± 0.2	—	73.9	0.0086 ± 0.0009	0.3	8.7 ± 1.5	19.8
G0	79 ± 0.5	—	72.0	0.16 ± 0.01	2.2	6.7 ± 1.0	12.4
G4	20 ± 0.3	—	68.6	0.15 ± 0.00	2.2	28 ± 8	25.0
I1	150 ± 0.7	—	72.8	—	—	5.4 ± 1.0	6.0
J1	12 ± 0.2	—	51.5	0.035 ± 0.002	4.5	3.5 ± 0.3	4.9
J3	24 ± 0.3	—	60.9	0.11 ± 0.01	9.7	16 ± 1	20.2
Cruise: F13OS/4 (Jan. 2014)							
B3	99 ± 0.6	—	62.8	4.8 ± 0.2	23.7	133 ± 22	51.1
E1	71 ± 0.5	—	65.0	0.39 ± 0.02	5.8	14 ± 4	18.5
E5	7 ± 0.2	—	64.5	0.23 ± 0.01	0.7	139 ± 19	65.2
G0	120 ± 0.6	—	61.7	0.63 ± 0.02	5.1	15 ± 2	20.2
G4	18 ± 0.3	—	64.6	0.24 ± 0.01	3.6	46 ± 13	30.3
I1	180 ± 0.7	—	64.0	1.1 ± 0.0	12.5	20 ± 3	18.0
J1	9 ± 0.2	—	52.7	0.057 ± 0.003	0.7	7.9 ± 0.6	11.3
J3	29 ± 0.3	—	60.2	1.2 ± 0.0	2.9	136 ± 10	52.5
Cruise: F14OS/1 (May 2014)							
B3	62 ± 0.4	2.08 ± 0.03	52.0	0.10 ± 0.003	4.7	3.6 ± 0.6	6.8
E1	40 ± 0.4	2.15 ± 0.03	71.1	0.024 ± 0.001	0.1	1.7 ± 0.5	3.6
E5	5 ± 0.2	4.38 ± 0.02	61.3	0.0032 ± 0.0002	0.1	2.4 ± 0.4	7.1
G0	130 ± 0.7	1.59 ± 0.08	33.3	0.29 ± 0.36	24.4	3.4 ± 4.2	12.1
G4	—	2.62 ± 0.04	39.4	—	—	—	—
I1	—	2.07 ± 0.04	35.9	—	—	—	—
J1	8 ± 0.2	1.17 ± 0.02	48.5	0.022 ± 0.001	0.7	3.1 ± 0.2	5.2
J3	—	3.30 ± 0.08	60.3	—	—	—	—
Cruise: F14OS/2 (Aug. 2014)							
B3	73 ± 0.5	2.02 ± 0.001	46.9	0.19 ± 0.01	7.9	5.2 ± 0.9	9.5
E1	140 ± 0.6	1.33 ± 0.10	35.8	0.35 ± 0.01	60.9	3.6 ± 1.0	8.7
E5	4 ± 0.1	2.60 ± 0.12	44.9	—	—	2.6 ± 0.6	6.6
G0	98 ± 0.6	1.69 ± 0.08	37.5	0.24 ± 0.01	14.5	4.1 ± 0.6	9.4
G4	—	1.88 ± 0.20	55.1	—	—	—	—
I1	—	1.93 ± 0.07	46.3	—	—	—	—
J1	16 ± 0.2	1.22 ± 0.02	55.9	0.016 ± 0.0003	1.1	1.3 ± 0.1	3.2
J3	—	2.71 ± 0.04	44.5	—	—	—	—
Cruise: F14OS/3 (Nov. 2014)							
B3	61 ± 0.5	1.59 ± 0.01	29.5	0.51 ± 0.02	13.0	11 ± 2	25.4
E1	43 ± 0.4	1.95 ± 0.03	35.2	0.14 ± 0.03	4.5	4.5 ± 1.5	12.5
E5	6 ± 0.2	3.12 ± 0.08	44.9	0.029 ± 0.003	0.8	13 ± 2	36.1
G0	96 ± 0.6	1.91 ± 0.05	37.0	0.18 ± 0.03	6.5	3.2 ± 0.6	11.0
G4	—	2.93 ± 0.06	45.3	—	—	—	—
I1	—	1.96 ± 0.02	36.3	—	—	—	—
J1	7 ± 0.2	1.30 ± 0.04	59.8	0.015 ± 0.001	0.7	3.1 ± 0.2	11.8
J3	—	2.90 ± 0.08	53.2	—	—	—	—
Cruise: F14OS/4 (Jan.-Feb. 2015)							
B3	53 ± 0.4	1.86 ± 0.08	48.4	2.46 ± 0.43	94.2	98 ± 23	66.9
E1	56 ± 0.4	1.64 ± 0.04	45.1	2.05 ± 0.19	60.6	66 ± 19	63.3
E5	4 ± 0.1	3.26 ± 0.07	60.8	0.013 ± 0.002	0.6	12 ± 2	43.5
G0	96 ± 0.6	2.11 ± 0.06	57.0	0.62 ± 0.03	8.4	17 ± 2	24.7
G4	—	3.12 ± 0.08	60.4	—	—	—	—
I1	—	2.11 ± 0.07	48.7	—	—	—	—
J1	8 ± 0.2	1.27 ± 0.14	51.4	0.21 ± 0.01	1.5	33 ± 3	23.1
J3	—	2.89 ± 0.11	56.0	—	—	—	—

Table S6. Dry weight and total radioactivity of ^{137}Cs in zooplankton samples.

Stn.	Dry weight (g-dry)	Total radioactivity of ^{137}Cs (Bq/sample)
Cruise: F12OS/1 (May 2012)		
B3	19.9	0.06
E1	116.0	0.20
E5	90.1	0.16
G0	51.7	1.11
G4	105.7	0.63
J1	6.3	1.17
J3	48.6	1.03
Cruise: F12OS/2 (Aug. 2012)		
B3	181.1	0.22
E1	63.6	—
E5	10.9	—
G0	94.7	0.31
G4	96.9	1.58
J1	71.0	0.68
J3	44.1	0.13
Cruise: F12OS/3 (Oct.-Nov. 2012)		
B3	116.3	0.17
E1	87.0	0.54
E5	124.8	—
G0	79.9	1.04
G4	128.2	0.63
J1	181.4	2.65
Cruise: F12OS/4 (Jan. 2013)		
B3	40.8	1.23
E1	49.5	2.87
E5	15.9	0.05
G0	24.0	0.21
G4	21.3	0.05
J1	58.9	2.98
J3	27.8	0.16
Cruise: F13OS/1 (May 2013)		
B3	67.0	0.05
E1	172.3	0.04
E5	292.8	0.08
G0	141.4	0.28
G4	171.9	0.06
I1	217.9	0.22
J1	112.5	0.29
J3	209.2	0.09
Cruise: F13OS/2 (Aug. 2013)		
B3	80.0	0.14
E1	55.8	0.17
E5	68.5	0.07
G0	50.5	0.16
G4	51.0	0.09
I1	71.9	0.63
J1	359.9	1.48
J3	101.4	0.07

Table S6 (continued)

Stn.	Dry weight (g-dry)	Total radioactivity of ^{137}Cs (Bq/sample)
Cruise: F13OS/3 (Nov. 2013)		
B3	23.3	0.02
E1	133.4	0.30
E5	48.5	0.12
G0	82.3	0.60
G4	38.4	0.27
I1	50.1	—
J1	127.1	0.10
J3	67.8	0.08
Cruise: F13OS/4 (Jan. 2014)		
B3	31.5	0.64
E1	11.8	0.08
E5	3.4	0.11
G0	19.9	0.25
G4	8.3	0.05
I1	12.5	0.11
J1	21.8	0.19
J3	5.9	0.24
Cruise: F14OS/1 (May 2014)		
B3	260.7	0.54
E1	242.6	4.83
E5	256.7	0.76
G0	172.8	0.21
J1	272.1	0.92
Cruise: F14OS/2 (Aug. 2014)		
B3	104.4	0.25
E1	87.0	0.05
E5	84.5	—
G0	99.4	0.16
J1	232.0	0.36
Cruise: F14OS/3 (Nov. 2014)		
B3	48.5	0.19
E1	109.3	0.33
E5	83.4	0.30
G0	94.2	0.26
J1	71.7	0.16
Cruise: F14OS/4 (Jan.-Feb. 2015)		
B3	28.4	0.07
E1	20.5	0.07
E5	12.3	0.03
G0	6.6	0.05
J1	1.2	0.02
